# Predation and Risk Behaviors of Free-Roaming Owned Cats in Auckland, New Zealand via the Use of Animal-Borne Cameras

**DOI:** 10.3389/fvets.2019.00205

**Published:** 2019-07-02

**Authors:** Stephanie J. Bruce, Sarah Zito, M. Carolyn Gates, Glenn Aguilar, Jessica K. Walker, Nick Goldwater, Arnja Dale

**Affiliations:** ^1^Royal New Zealand Society for the Prevention of Animal Cruelty, Auckland, New Zealand; ^2^Research and Postgraduate Centre, Unitec Institute of Technology, Auckland, New Zealand; ^3^School of Veterinary Sciences, Massey University, Palmerston North, New Zealand; ^4^Environmental and Animal Sciences, Unitec Institute of Technology, Auckland, New Zealand; ^5^New Zealand Companion Animal Council, Auckland, New Zealand; ^6^Wildland Consultants Ltd., Auckland, New Zealand

**Keywords:** owned cat, free-roaming cat, predation, risk behavior, cat behavior, native fauna species, welfare, cat management

## Abstract

Free-roaming cats are at increased risk of injuring themselves as well as other domestic and fauna species, yet relatively little is known about the frequency at which risk and predation behaviors occur in a typical day. In this study, cat risk, and predation behavioral information was collected using animal-borne video cameras and global positioning system (GPS) units that were attached to break-free cat collars. The observation period was one to three consecutive days for 37 convenience sampled free-roaming owned cats in Auckland, New Zealand. Video footage was manually reviewed and all predation and risk behavior events were recorded. These included stalking, pursuing, and seizing prey as well as altercations with other cats, ingesting harmful substances, and venturing into hazardous locations such as roads and storm drains. During the observation period, 23 of the 37 cats (62.2%) engaged in a total of 121 predation events. Of these, 40 resulted in successful prey capture with 18 of the 40 captures involving New Zealand native fauna species. Invertebrates were the most common taxa preyed upon (*n* = 55; 46%), followed by skinks (*n* = 8; 7%). No mammalian, avian or amphibian prey were captured and no cat took prey back to their residence. A total of 326 risk behaviors were observed for 32 out of the 37 cats (86.5%) with the most common being cats venturing onto the road (*n* = 132; 41%). Younger cats (aged 1–6 six years) engaged in significantly more predation and risk behaviors than older cats (aged 7 years and above). Sex, breed, number of cats in a household, and geographic location were not found to be predictors of cats' participation in predation or risk behaviors. Given the high frequency of predation and risk behaviors in free-roaming owned cats, it may be beneficial to educate owners about strategies to minimize risk such as housing them indoors, containing them to their properties or monitoring their time spent outdoors.

## Introduction

The New Zealand Animal Welfare (Companion Cats) Code of Welfare 2007 defines companion cats (*Felis catus*) as domestic cats that cohabitate with humans and depend on humans for their welfare (1). Internationally, this category of cats is generally referred to as “owned” and this term will be used throughout the rest of this paper. Domestic cats are commonly kept in New Zealand, with ~1,134, 000 owned across the country (2). An estimated 90% of New Zealand's owned cats can free-roam without being monitored by their owners, having access to the outdoors during the day, night, or always (3), compared with 97% in Britain (4), 80% in Sydney, Australia (5), and 64–76% in the USA (6). When allowed to roam freely, companion cats are capable of hunting fauna species and engaging in behaviors that may cause themselves harm, such as fighting with other cats, ingesting harmful substances, and running onto roads (7). Consequently, there is growing interest in developing recommendations for owners to mitigate these risks.

It is important to protect native fauna species for the benefit of New Zealand's biodiversity. Cats can kill a range of species, with mammals, birds, invertebrates, and reptiles reported as being common prey taxa brought home by owned cats in New Zealand (8–10), many of which include New Zealand native fauna species (11). New Zealand native fauna is particularly vulnerable to predation by mammalian predators such as cats, given that it evolved in the absence of mammalian quadrupeds (12, 13). For example, van Heezik et al. (10) reported that the survival of three bird populations in urban Dunedin, New Zealand, was negatively affected by owned cat predation, including those of two native species (fantail [*Rhipidura fuliginosa*] and bellbird [*Anthornis melanura*]). However, cats are also recognized as being potential population regulators of other introduced predators and competitors, controlling the number, and subsequently the predation impacts of these species (14). This highlights the complexity of the situation, with a possible positive effect of cats controlling introduced species that can threaten native species. It has been suggested that cat management should be conducted in tandem with the management of other introduced species to avoid a surge in the numbers and, therefore, hunting rates of other predatory species that cats prey upon, such as rats (*Rattus* spp.) and mice (*Mus musculus*) (8, 14). This phenomenon, in which populations of medium-sized predators rapidly increase in ecosystems after the removal of larger carnivores, is known as “mesopredator release,” and can potentially lead to adverse effects on the ecosystem (15).

Venturing onto the road, fighting with other cats, and consuming potentially toxic solids and liquids are examples of common risk behaviors associated with free-roaming cats that can result in distress, injury, disease, and/or death (16–19). The longevity of owned cats varies among studies: reported as a median of >12.5 years in purebred insured cats in Sweden (20), mean of 8.4 ± 5.6 years in Taiwan (21), median of 14.0 years in the UK (22), and an “average” longevity of 12.1 years in the USA (whether this was a median or a mean value was not specified). Both the UK and Swedish studies suggested the existence of two distinct subpopulations of cats: those with a propensity for earlier death (with a large number of road traffic accident-related deaths among these younger cats) and cats that survive to an older age (20, 22). Although these studies did not discuss outdoor access as a risk factor, it is likely that the cats who died due to road traffic accident-related mortality had outdoor access. This highlights one of the considerable risks to which free-roaming owned cats are exposed. The different attitudes to outdoor access for owned cats may affect trauma-related mortality and longevity; daily outdoor access has been reported to vary from over 90% of UK cats to 80% of Australian cats, and 50–60% of cats in the USA (5, 23, 24).

Cat owners may be aware of some dangers their cats face whilst free-roaming, but may not be aware of other risks. For example, venturing onto the road and climbing into storm drains or on the edges of roofs (19). It has been theorized that cat owners may be more likely to engage in cat management methods where they are made aware of the benefits to their cat's welfare, in contrast to engaging in cat management simply out of concern for the preservation of fauna species (19, 25). By being informed about the danger of allowing their cats to free-roam owners could be encouraged to manage their cats more closely by housing them indoors, containing them to their properties, or monitoring their time outdoors. This will likely benefit the welfare of their cats as well as help reduce predation impacts on native fauna species.

Many factors may influence a cat's engagement in predation, the species of animal they prey upon, and their risk behaviors. These include age, sex, location (for example, urban, suburban, or rural), provision of outdoor access, breed, time of day, provision of food, and the number of owned cats in the household. Age is suggested to be a predictor of predation behavior in some studies with younger cats reported to engage in hunting more than older cats (26), although age has not been found to influence predation rates in other studies (27). Sex and breed have not been shown to be predictors of hunting rates (26–28) but are predictors of cats venturing onto the road. For example, male cats and non-pedigree cats are reportedly more likely to venture onto roads and be involved in road accidents than female and pedigree cats (16, 19). Cats living near populations of native fauna species (a situation that may be more common in rural and suburban areas than in urban areas) will presumably capture more native prey than other cats (27, 29). These are contradictory ideas that, to date, have not been tested. Cats housed indoors at night engage in fewer risk behaviors and may capture fewer prey items than those cats allowed outside all day and night (19). However, Rochlitz (17) found that cats kept indoors at night were just as likely to be involved in a road accident as cats always provided outdoor access. The presence of other owned cats in a household may facilitate (24, 30) or hinder (31) engagement in predatory behaviors, and so too may the absence of other cats (32).

The current study investigated owned cat risk and predation behavior using animal-borne cameras. To date, no research into the predation and risk behaviors of owned cats employing animal-borne technology has been conducted in New Zealand. Previous studies in this country have used alternative methods to determine the prey items of owned cats' prey, such as owner survey methodologies to investigate prey taken back to an owned cat's residence, stomach content analysis and scat analysis (9, 10, 33). Whilst these methods provide insight into the predation behaviors of cats, information regarding whether any prey was scavenged or lost/left *in-situ* is unable to be collected (34, 35).

The use of animal-borne video cameras allows this information to be collected and can provide a more accurate depiction of cat predation behaviors than other methods. To the authors' knowledge, no research has been undertaken in New Zealand to identify or quantify the extent to which free-roaming cats engage in risk behaviors. The current study aimed to better understand free-roaming owned cats in Auckland, New Zealand, in terms of their engagement in predation and risk behaviors, home ranges, and activity levels^1^ via the use of animal-borne camera and global positioning system (GPS) technologies. The factors that may act as predictors of these behaviors were also investigated. It is anticipated that this research could be used to assist in determining how owned cats can be managed, both for the protection their welfare and that of native fauna species.

## Materials and Methods

### Participant Selection

Cat owners for the study were recruited via an all-staff e-mail listserv advertisement at Unitec Institute of Technology sent on October 30th 2016 and via two advertisements, ~1 month apart, on the New Zealand Companion Animal Council Facebook page. The advertisements provided a brief description of the research and, as an incentive to participate, owners were offered the chance to view a selection of their cat's footage upon completion of the research. To ensure interested owners knew and agreed with what the research entailed, each was emailed a research participant information sheet that outlined the research in full as well as a consent form which required their signature. Interested owners were also asked a series of questions to confirm that their cat(s) met the following study inclusion criteria:

- The cat lived in the Auckland Region.
- The cat was over 6 months of age to facilitate their adaption to carrying the weight of the camera.
- The cat had access to the outdoors (i.e., not an indoor only cat).
- The cat was classified as an owned cat (1).
- The cat was able to be safely handled by their owner.
- The cat was seen/handled by their owner every day.

Seventy-two cat owners responded to the advertisements. Of these, 35 owners with a total of 51 cats met the eligibility criteria. Due to study resource limitations, the 29 owners that contacted the research team first were selected to participate in the research study, totalling 41 owned cats. Nine households had multiple cats participate. However, with technological issues and one cat rejecting wearing the camera, useable footage was collected from 37 of the 41 participating cats, representing 26 households across Auckland. With the small pool of eligible cats from a potentially non-representative sample of owners, we focused on characterizing the range of predation and risk behaviors among free-roaming owned cats rather than trying to make accurate inferences about the true prevalence of these behaviors across all free-roaming owned cats.

Ethical approval for this research was obtained from the University of Auckland AEC (Auckland, New Zealand; Reference 001595).

### Technology

Video footage to observe cat engagement in predation and risk behaviors was collected using KittyCam^©^ animal-borne cameras (National Geographic, Washington, D.C., USA) and GPS data were collected using animal-borne GPS units (Petrek, Auckland, New Zealand). KittyCams^©^ are part of National Geographic's Crittercam^©^ series and have been developed specifically for use on domestic cats. Crittercams^©^ have been used in numerous studies since their conception in 1987, investigating the behaviors of a variety of both aquatic and terrestrial animal species, including domestic cats (7, 19, 36, 37). KittyCams^©^ are rectangular, waterproof units that weigh 90 g each. These units were attached to a break-free cat collar (“AlleyCat” in size small; Rogz, Cape Town, South Africa) with cable ties and sat underneath the cat's chin, collecting video footage from their point of view (19). An infrared light-emitting diode (LED) positioned next to the KittyCam^©^ camera lens allowed for recording in darkness. In-built motion sensors prompted the KittyCam^©^ to record when cats were moving and stop when they were not moving, conserving battery power and memory card space. KittyCams^©^ had the capacity to record 10–12 h of footage before requiring charging. Programmable settings included the timing of activation and deactivation of the KittyCam^©^, duration of recording once motion ceased and the intensity of movement required before recording was initiated. Each KittyCam^©^ had an internal very high frequency (VHF) transmitter for use in locating missing units.

GPS data were collected using waterproof Petrek^©^ GPS units (Petrek, Auckland, NZ). Each unit weighed 30 g, and was attached to the back of the break-free collars. To pinpoint and record a cat's location the GPS units used cell phone networks and sat on the back of a cat's neck. GPS accuracy ranged from 0.5 to 30 m+ based on signal strength and quality. The GPS units updated each cat's location every 5 min or when a signal was available and required charging at 24 h intervals. The GPS capability was used as the primary method of assisting with the location of missing collar sets (i.e., a collar with one camera and one GPS unit attached). The GPS data on cat movements and home ranges will be presented in another manuscript.

The break-free collars were set to the highest load setting to accommodate the weight of the cameras and GPS units. Each collar came equipped with a bell, which was removed because bells have, in the study of Gordon et al. (28), been shown to alert prey items to a cat's presence, potentially reducing the capture of birds by 50% and rodents by 61%. In contrast, bells on cat collars were not shown to significantly reduce the amount of prey captured by cats in the study by Morgan et al. (38). VHF telemetry equipment (Sirtrack^®^ receiver—R-1000 Telemetry receiver, antenna−3 element folding yagi antenna) was used as a secondary method of locating missing cameras where they could not be found using the GPS units.

### Experimental Protocol

Collar sets were deployed on pre-arranged dates between 1st November 2016 and 11th April 2017.

Owned cats wore one collar set per 24-h period with the intention of collar sets being changed after each period for three consecutive days. A 3-day recording period per owned cat was chosen because it represented a balance between sample size and the amount of data collected per cat, based upon resource availability.

Cat owners were taught to attach the collars to the cats to minimize the potential distress associated with being handled by an unfamiliar person. Owners were instructed to remove the collars if they had concerns about the cats' welfare. Inability to adjust to the collars may have caused the cats distress, potentially impacting their welfare and the accuracy of the data collected (39, 40). Collar sets were collected upon completion of the data collection period. Video footage was downloaded and the KittyCams^©^ and GPS units were prepared for successive deployments. A maximum of two cats wore collars at one time to be logistically manageable for the research team. If a collar was lost from a cat during the recording period, another collar was not attached to reduce the risk of losing further equipment. Although the GPS data are not reported in this paper, participating cats were wearing GPS devices at the same time as the KittyCams and this detail has been included to improve the reproducibility of the study and to allow discussion about weight of the cameras and GPS units that may have impacted the participating cats' behavior.

During the researcher's visit, cat owners were asked the following information about their cats: age, sex, breed, sterilization status (sterilized or entire), location (rural, suburban, and urban), when the cats were let outside (outdoors all the time, inside at night, inside sometimes), whether or not they had worn a collar before (yes, no, unsure), and whether it was a multi-cat household (yes or no). Information on temperature (deg C) and weather conditions (dry, light rain, or heavy rain/thunderstorms) was collected from the MetService website, as these factors may have influenced the likelihood of cats spending time outdoors and engaging in predatory or risk behaviors on any given day. Cat age was categorized into two groups: 1–6 years and 7 years and above. These groupings allowed for comparison with the findings of Morgan et al. (38), who used similar age groups. Breed was categorized into domestic (including domestic long hair, domestic medium hair, and domestic short hair) and other breeds.

### Data Processing

For various reasons including technological malfunction and one cat refusing to wear the collar set, video footage was only able to be collected from 37 owned cats. Altogether, 22 cats (48%) were observed for 3 days, nine cats (20%) were observed for 2 days, and 15 cats (33%) were observed for 1 day for a total of 99 observation days. A total of 179.8 h of footage was collected.

The video footage was reviewed manually by the research team to characterize the frequency and duration of predation events and risk behaviors during the observation period. Predation events were defined as when the footage indicated a cat was stalking, pursuing, or seizing prey items (defined as all the animals that cats were observed attempting to capture or successfully capturing). Similar definitions have been described previously by Loyd et al. (7) and McGregor et al. (41). All predation behaviors were documented, including those that resulted in unsuccessful prey capture and instances of scavenging. Risk behaviors that participating cats were likely to display were defined prior to data collection, being modeled on those presented by Loyd et al. (7). These behaviors included “altercations with other cats,” “venturing onto the road,” “climbing underneath car,” “ingesting solids not provided by owner/carer,” “ingesting liquids not provided by owner/carer,” “climbing on edge of roof,” “climbing into storm drain,” and “other.” All observed risk behaviors were recorded.

The KittyCam^©^ internal motion sensors provided a simple method of determining the total amount of time cats were active whilst wearing the collars. Daytime footage for each cat (i.e., that collected between 6 a.m. and 6 p.m.) was combined to determine the amount of time they spent active during the day; night time activity levels were determined in a similar fashion.

### Statistical Analysis

Basic descriptive statistics were provided on the demographic characteristics of the cats included in the study population as well as the frequency and characteristics of the predation and risk behavior events. To evaluate factors influencing predation and risk behavior, two binary outcome variables were created for each cat observation day: had at least one predation event (yes or no) and had at least one risk behavior event (yes or no). Mixed-effects logistic regression models with individual cat as the random effect were then used to evaluate the following risk factor variables: age (under 6, 7 years, or older), sex (male or female), breed (domestic or other), season of year (winter, spring, summer, or fall), weather (sunny, light rain, heavy rain/thunderstorms), whether the cat had previously worn a collar (yes or no), location (urban, suburban, or rural), and when allowed outside access (all the time or partial day). Although we attempted running a mixed-effects negative binomial with counts as the outcome, the models would not converge, likely due to the relatively small sample size and so we chose the more conservative mixed-effects logistic regression to account for the repeated measures in individual cats. An initial univariable screen was performed to identify factors that were associated with the outcome of interest with a *p*-value of < 0.20 for inclusion in the multivariable model. As only one variable reached significance, a multivariable analysis was not performed. The results were reported as odds ratio (ORs) with 95% confidence intervals. All statistical analysis were performed in the R statistical software package (42).

## Results

### Cat Demographics

Owned cats were aged between 1 and 13 years; mean 6.7 years (±4.0) (Tables 1, 2). Eighteen owned cats (49%) were male (Tables 1, 2). Nineteen owned cats (51%) were female, all cats were sterilized (100%) (Tables 1, 2).

**Table 1:** Summary of cat demographics.

**Variable**	**Categories**	**Number (%) of owned cats**
Age (years)	0–6	18 (49%)
	7+	19 (51%)
	Unknown	0 (0%)
Sex	Male	18 (49%)
	Female	19 (51%)
Location	Urban	5 (14%)
	Suburban	26 (70%)
	Rural	6 (16%)
Outdoor access	At all times	24 (65%)
	Inside at night	13 (35%)
Multi-cat household	Yes	20 (54%)
	No	16 (43%)
	Unknown	1 (3%)
	6+	n/a
Breed	Non-pedigree	27 (73%)
	Pedigree	10 (27%)
Number of days of footage recorded	1	22 (60%)
	2	9 (24%)
	3	6 (16%)

**Table 2:** Individual cat demographics.

**Cat**	**Age (years)**	**Sex**	**Location**	**Outdoor access**	**Multi-cat**	**Breed**	**No. of days of footage collected**
1	2	Male	Rural	At all times	Yes	Burmese	3
2	2	Male	Rural	At all times	Yes	Burmese	3
3	2	Female	Suburban	At all times	No	DSH	3
4	6	Female	Rural	At all times	Yes	Burmese	3
5	11	Female	Suburban	Inside at night	No	DSH	3
6	4	Male	Suburban	Inside at night	Yes	DSH	3
7	3	Female	Urban	At all times	Yes	DSH	2
8	3	Male	Rural	At all times	No	Siamese	2
9	13	Male	Rural	At all times	No	DSH	2
10	8	Male	Suburban	Inside at night	Yes	Burmese	2
11	13	Male	Suburban	Inside at night	Yes	DSH	3
12	12	Female	Suburban	At all times	No	DSH	1
13	1	Male	Suburban	Inside at night	No	DSH	2
14	11	Male	Suburban	At all times	No	DSH	3
15	7	Female	Suburban	Inside at night	No	DSH	3
16	3	Female	Rural	At all times	Yes	DLH	3
17	11	Female	Urban	At all times	Yes	Russian blue	3
18	3	Male	Suburban	Inside at night	No	DMH	2
19	2	Female	Suburban	Inside at night	No	DSH	1
20	7	Female	Suburban	Inside at night	Yes	DSH	3
21	8	Female	Suburban	Inside at night	No	DSH	3
22	11	Female	Suburban	At all times	No	DSH	3
23	1	Male	Urban	At all times	No	Norwegian forest cat	3
24	2	Male	Suburban	Inside at night	Yes	Burmese	1
25	4	Male	Suburban	At all times	Yes	DSH	3
26	5	Female	Suburban	At all times	Yes	DSH	2
27	10	Male	Suburban	At all times	Yes	DSH	3
28	10	Female	Suburban	At all times	Yes	DSH	3
29	9	Male	Suburban	Inside at night	No	DSH	3
30	4	Female	Suburban	At all times	No	DSH	3
31	6	Female	Suburban	At all times	No	DMH	2
32	9	Female	Urban	At all times	Yes	Russian white	3
33	12	Male	Urban	At all times	Yes	DSH	3
34	12	Female	Suburban	At all times	Yes	DSH	1
35	10	Female	Suburban	At all times	Yes	DSH	2
36	2	Male	Suburban	At all times	No	DLH	1
37	8	Male	Suburban	Inside at night	Unknown	Bengal	1

### Predation Results

During the 90 observation days for owned cats, there were 121 predation events. Owned cat predation events ranged from 2 s to 6 min in length, with a mean of 35.5 s (±44.07). Forty (33%) of the 121 owned cat predation events resulted in successful prey capture, 56 (46%) in unsuccessful capture, 22 (18%) in undetermined success, and three (3%) in scavenging (Table 3). Invertebrates were the most common taxa preyed upon by owned cats (*n* = 55; 46%), followed by skinks (*n* = 8; 7%). Owned cats did not capture any mammalian, avian or amphibian prey, though an already deceased unidentified bird was scavenged (Table 4). Fifty-seven (47%) prey items hunted by owned cats could not be identified to phylum level (Table 4). Twelve owned cat predation events (0.1%) resulted in the successful capture of native species (Table 5).

**Table 3:** Fate of successfully captured prey items.

**Prey fate**	**Count**
Killed and fully or partially consumed	33
Captured and released	5
Killed and left *in-situ*	1
Unknown	1
Total	40

**Table 4:** Prey identification by taxa.

**Species**	**Count**
**Invertebrates**	
Wētā^*^	16
Blowfly (Calliphoridae)	1
Unidentified fly	1
Cicada^*^ (Cicadidae)	13
Huhu beetle^*^ (*Prionoplus reticularis*)	1
Cricket^*^ (Gryllidae)	1
Cellar spider (Pholcidae)	1
Unidentified moth	1
Praying mantis (Mantodea)	4
Monarch butterfly^*^ (*Danaus plexippus*)	1
**Reptilian**	
Plague skink (*Lampropholis delicata*)	7
Copper skink^*^ (*Oligosoma aeneum*)	1
**Avian**	
Unidentified bird	1
Unidentified insect	15
Unidentified	57
Total	121

* *Indicates a New Zealand native species*.

**Table 5:** Predation event outcome by prey species.

**Successful**	**Count**	**Not successful**	**Count**	**Unknown success**	**Count**	**Scavenged**	**Count**
Wētā^*^	11	Weta	5	Blowfly	1	Cicada^*^	1
Unidentified fly	1	Cellar spider	1	Cicada^*^	1	Unidentified bird	1
Plague skink	3	Plague skink	4	Monarch butterfly^*^	1	Unidentified insect	1
Cicada^*^	10	Copper skink^*^	1	Unidentified insect	1		
Huhu beetle^*^	1	Cicada^*^	1	Unidentified	18		
Cricket^*^	1	Unidentified moth	1				
Unidentified insect	7	Praying mantis	4				
Unidentified	6	Unidentified insect	6				
		Unidentified	33				
Total	40	Total	56	Total	22	Total	3

* *Indicates a New Zealand native species*.

### Risk Behavior Results

During the 90 observation days for owned cats, there were 326 risk behavior events recorded. The incidence of each risk behavior and the number of cats that participated in each risk behavior varied. The most common risk behavior observed was cats venturing onto the road (Table 6). Three altercations (27%) between owned cats that did not live together resulted in physical contact and eight (73%) did not, involving only growling, and swiping. Solids ingested that were not provided by owners included twigs, discarded food, and potted plants, while liquids included water from paddling pools, freshwater streams, puddles, and roof gutters. The counts of “other” risk behaviors witnessed included a cat climbing on Pink Batts^®^ (glass wool home insulation).

**Table 6:** Risk behaviors displayed by study cats.

**Behavior**	**Count**	**No. of cats involved**
Altercations with other cats	11	5
On road	132	12
Climbing underneath car	3	2
Ingesting solids not provided by owner	33	15
Ingesting liquids not provided by owner	98	22
Climbing on the edge of roof	40	8
Climbing into storm drain	1	1
Other	8	1
Total	326	

### Factors Influencing Predation and Risk Behaviors

There was considerable variability within and between cats in both the number of predation events and the number of risk behavior events observed on any given day. The maximum number of predation and risk behavior events observed in 1 day were 10 and 25, respectively. Table 7 shows the daily counts of predation events and risk behavior events for the 22 cats with three complete observation days. While most owned cats had a relatively low number of events, some cats were clearly more active than others. There were 36/99 (36.4%) cat observation days with at least one predation event and 74/99 (74.7%) cat observation days with at least one risk behavior event to include in the mixed-effect logistic regression models. For predation events, age was the only significant predictor. Cats that were over 7 years of age were 0.20 times as likely to have at least one predation event compared with cats 6 years of age and under (OR: 0.20, 95% CI 0.09–0.42, *p* < 0.001). None of the variables in the model for risk behaviors achieved significance.

**Table 7:** Count of predation behaviors and risk behaviors per day for the 22 owned free-roaming cats with three full observation days.

	**Predation behaviors**				**Risk behaviors**			
**Cat**	**Day 1**	**Day 2**	**Day 3**	**Total**	**Day 1**	**Day 2**	**Day 3**	**Total**
1	6	8	0	14	7	5	2	14
2	0	0	2	2	11	0	1	12
3	4	0	1	5	0	0	0	0
4	1	0	2	3	1	3	5	9
5	0	0	0	0	3	0	5	8
6	2	6	9	17	2	8	18	28
11	0	3	2	5	0	5	6	11
14	2	0	0	2	6	0	5	11
15	0	1	0	1	0	2	1	3
16	1	0	10	11	4	1	3	8
17	0	0	0	0	4	3	3	10
20	0	0	0	0	0	0	1	1
21	1	0	0	1	5	3	1	9
22	0	0	0	0	2	2	0	4
23	0	0	0	0	2	4	9	15
25	0	3	1	4	25	18	9	52
27	2	0	0	2	2	0	1	3
28	0	2	0	2	0	7	1	8
29	0	0	0	0	2	5	0	7
30	4	0	0	4	14	4	8	26
32	0	1	0	1	6	5	2	13
33	0	0	0	0	1	2	2	5

### Activity Results

Owned cats recorded between 0.36 and 8.0 h of footage each in total, with a mean of 4.9 h (±2.4).

Owned cats spent 86.35% of the time inactive and 13.65% of the time active; hunting comprised 0.09% of active time and engagement in risk behaviors comprised 0.18%. The remaining time spent active included behaviors such as grooming, walking, and ingesting food and water provided by their owners.

## Discussion

This study reports the first observations of predation and risk behaviors of owned cats in New Zealand using animal-borne cameras and demonstrates that predatory and risk behaviors were commonly displayed by the cats.

Most of the identified prey species in the current study were invertebrates. No mammals, amphibians or birds were preyed upon and only one case of a bird being scavenged was observed. This is in contrast to studies that used owner survey methodologies to investigate prey taken back to an owned cat's residence in New Zealand. In these studies, it was reported that mammals or birds were most commonly taken back, followed by invertebrates and reptiles, with other prey species being taken infrequently, including amphibians and fish (9, 10, 33). Loyd et al. (7), using animal-borne camera technology, found that reptiles were successfully captured most frequently, followed by mammals, invertebrates, birds, and amphibians. This research closely resembles the results of the current study, suggesting that different methodologies may be a factor in determining prey composition data. A higher rate of prey identification in the current study may also have altered prey composition results.

Cats are opportunistic and generalist predators capable of killing a variety of prey species (7, 27, 43). Invertebrates and small reptiles may have been more abundant during the seasons in which data were collected and, consequently, were the easiest targets for predation by the opportunistic companion cats. This may explain why these species accounted for the majority of prey captured. Other possible explanations for the absence of mammalian and avian prey include individual cat prey preferences and a short data collection period which did not cover multiple seasons. It is possible that the seasons in which data were collected may affect prey abundance and availability (44–47). In addition, it has been suggested that an infrared LED, such as that next to the KittyCam^©^ camera lens, which allowed for recording in darkness, may influence the behavior of potential prey (37, 48). However, it allows information to be collected that would otherwise be missed (48). Prey activity patterns change during a 24-h period (27), likely influencing what cats hunt at different times of the day. Accordingly, to gain the most accurate representation of cat predation behaviors, it was necessary to use the LED.

New Zealand native fauna species comprised 15% of observed predation events and 30% of successful prey captures. Previous research using owner survey methods has suggested that native fauna species comprise 4 to 40% of owned cat prey (9, 10, 33). The capture of native species occurred in all locations (urban, suburban, and rural) and did not occur in one location significantly more than another. This is an interesting result given the common perception that cats living in rural areas or areas of ecological significance hunt native fauna species to a greater extent than cats living elsewhere (25, 49). The results of the current study suggest that popular opinion regarding cat predation behavior may not always be correct, and that the enforcement of management techniques based on cat location (e.g., cat exclusion zones) may not do enough to mitigate the depredation of native fauna species, if not accompanied by other management techniques. There was a low level of observed cat predation of huhu beetles (*Prionoplus reticularis*), copper skinks (*Oligosoma aeneum*) and wētā (e.g., *Hemideina* and *Hemiandrus* spp.) in this study. However, Huhu beetles are common in New Zealand forest habitats and copper skinks are common in the North Island of New Zealand (50–52). Captured wētā were not identified to species level, which meant the conservation status of the captured wētā was not determined.

No prey items were taken back to a cat's residence, indicating that predation information based exclusively on the prey items a cat takes home may greatly underestimate the amount of prey items captured by owned cats. Loyd et al. (7) drew the same conclusion upon observing that cats brought home only 23% of prey they captured. Underestimation of invertebrate capture may be especially common, with 31 invertebrates being captured and/or killed *in-situ* in the current study, 12 of which were native species. The capture data in this study were collected largely during warmer months when the abundance of some prey species, including invertebrates, has been found to be highest (44, 47). This in turn may have produced results that overestimate invertebrate predation, if extrapolated throughout the rest of the year. However, with ~300 New Zealand native terrestrial invertebrate species threatened with extinction (53), it is suggested that the effect of cat predation on the survival of invertebrate species should be the focus of future research. Targeted conservation efforts may be required to save native invertebrate species from extinction due to predation by animals, including cats but also other species such as rats (*Rattus rattus, Rattus norvegicus, Rattus exulans*), mice (*Mus musculus*), and hedgehogs (*Erinaceus europaeus*), protecting them in their role as regulators of healthy ecosystem functioning (54, 55).

Young cats captured more prey than older cats in this study, which supports the results of previous studies (9, 10, 26). This result suggests that management of younger cats could be prioritized over the management of older cats to more effectively reduce predation rates. In line with the findings of previous studies (26–28), sex, and breed did not appear to influence predation rates. However, the results from the statistical modeling must be interpreted with caution given the small sample size and difficulty fitting robust mixed-effects models to the data. Although not significant in our models, there have been varying hypotheses on how the number of cats in a home may influence predatory behaviors (24, 30–32). In future studies, it would also be useful to assess other cat demographic management factors such as feeding, sterilization status, health status, temperament, socioeconomic, and environmental characteristics of the neighborhood, and density of other free-roaming cats. These factors may influence both the likelihood of seeking and encountering prey as well as potential exposures to risk.

Five cats engaged in altercations with cats they did not live with, with three altercations resulting in physical contact between the cats. Cats may sustain wounds when fighting that can become infected or contract diseases transmitted by contact with carrier cats, such feline immunodeficiency virus (FIV) (56, 57), though lower rates of infectious diseases, including FIV, have been observed in sterilized cats than in non-sterilized cats (58–60). Venturing onto the road was the most common risk behavior that participating cats engaged in, putting them at risk of injury and/or death if they were hit by a vehicle. Of the 116 owned cats hit by vehicles that Rochlitz (61) collected data on over an 11-month period, 28 died because of the accident and most others sustained injuries ranging in severity from minor to life-threatening. Two owned cats climbed underneath and up into various parts of a car, including the wheel well. Whilst this behavior puts cats at risk of injury and death should they become trapped, it appears not to be as significant a risk to cat welfare as other behaviors witnessed in this study. A similar result was observed by Loyd et al. (19), with only one cat climbing into a car engine in their study.

Participating cats also frequently ingested plant material and water from potentially contaminated sources. Numerous common plants are toxic to cats, including lilies (*Lilium* spp.), aloe vera (*Aloe vera*) and daffodils (*Narcissus* spp.). Ingestion of these plants can result in vomiting, diarrhea, and kidney and cardiac failure (62). There is also a risk that cats, especially those living near areas of ecological significance, will consume poisons laid to kill invasive pest species. Sodium fluoroacetate (1080) is routinely used in New Zealand to kill mammalian pests such as brushtail possums (*Trichosurus vulpecula*), rats and stoats (*Mustela erminea*) (63). Free-roaming cats may encounter and ingest 1080 and become ill or die, given that the lethal dose for an average-sized adult cat is less than that for a possum (64). Given the propensity for cats to scavenge, sub-lethal doses of 1080 may be ingested if cats consume animals killed by the poison, resulting in vomiting, staggering and drowsiness before being excreted, with no long-term effects on health reported (64, 65). Cats that consume water from puddles may inadvertently ingest toxins such as car coolants and oils, insecticides, and pesticides, which can result in sickness or death (19, 66–68).

Cats were often observed climbing on the edges of house, shed or garage roofs, and one owned cat was seen climbing into a stormwater drain. These behaviors put cats at risk of serious injury or death should they fall from a roof or get trapped in a drain. Twenty percent of the cats in the study of Loyd et al. (19) climbed on roofs and in trees, a similar percentage to that of the current study, suggesting that this is a common risk behavior that cat owners should be aware of Loyd et al. (19) also witnessed 20% of the cats in their study climbing into storm drains—a far higher percentage than that of the current study—indicating that this may be more of a concern for owners in the USA where their study was conducted, possibly due to the increased ease in which cats can access the drain systems there. Risk behaviors classed as “Other” involved one owned cat climbing on Pink Batts^®^ insulation, which can cause minor cuts and skin irritation as well as respiratory issues if inhaled or ingested. Whilst not commonly observed, these risk behaviors highlight the range of risks that free-roaming cats routinely encounter.

The sterilization status of cats may influence their behavior; reduced aggression has reported in sterilized stray female cats compared to entire stray female cats (69). In addition, roaming, fighting, and aggressive behaviors can be associated with higher risk of injury and infectious disease (20, 70, 71). Aggression, fighting, and roaming have been reported to decrease after sterilization (72, 73). All participating cats were sterilized; consequently, the influence of sterilization status on companion cat predation and risk behaviors, and activity levels could not be determined in the current study. Little conclusive research has been reported on whether sterilization status impacts on cat predation and risk behaviors, and activity levels. Nonetheless, the majority of companion cats are reportedly sterilized in New Zealand: companion cat sterilization levels have been reported to be as high as 90% in Auckland, New Zealand (74) and 93% nationwide (2). Therefore, the overall behavior of companion cats is considered unlikely to differ substantially from that found in the current study.

The current study is the first of its kind in New Zealand and it would be useful to replicate the study in other parts of the country outside of Auckland to determine whether the results are applicable on a nationwide scale and to further explore animal characteristics that may be influencing behavior. We acknowledge that our study population was small and that the owners were potentially non-representative due to voluntary response bias. However, as our objective was to characterize the range of predation and risk behaviors exhibited by cats rather than estimate the true prevalence, there were likely enough data to achieve information saturation (75). It is unclear why no mammalian or avian species were captured in the current study. It is possible that the weight or novel feeling of the camera may have disrupted cats' normal behaviors and subsequently their prey choice (76). However, it is noted, that Loyd et al. (7) observed cats capturing mammals and birds whilst wearing the same cameras. The added weight of the GPS unit may have been a determinant in the disruption of cat behavior and prey choice, with Coughlin and van Heezik (76) observing that cats behaved differently when a “heavy” device (136 g) was worn compared to a “light” device (36 g). For the majority of cats, we were only able to obtain footage from a single observation day and it was therefore difficult assess whether the patterns of behavior were likely to remain consistent over time.

It is suggested that in future research, sequential assessments of the same cats over time are performed and it may also be beneficial to “train” participant cats to wear the monitoring gear prior to capturing data. It is also recommended that future research collect data across all four seasons to ascertain the effect that changes in prey abundance has on overall predation rates and prey composition. The climate in Auckland is subtropical, with the weather being characterized by mild winters and relatively warm and humid summers (77). Spring and autumn are mild with more rainfall experienced in spring than in autumn. Average temperatures between summer and winter vary less than in other countries, fluctuating by no more than 14°C (77). Cats may be less likely to roam over winter months when the weather is less hospitable (78), although no seasonal variation was found in a study in Perth, Australia (79), and it was found in another study conducted in Christchurch, New Zealand that companion cats were more active in a wetland during winter rather than in summer (80). It is possible that a smaller home range size may influence predation and risk behaviors. With the limited resolution of the camera footage, there were some difficulties in accurately identifying prey items and so it is possible that the distributions reported in this study do not reflect the true distribution of species that cats routinely prey on.

The information presented here could be used to educate cat owners on the welfare advantages of managing their cats more closely, i.e., by housing them indoors, containing them to their properties using cat enclosures and containment systems, or monitoring their time spent outdoors. Education material (verbal, reading material, posters/videos in waiting room) could be provided by veterinarians when animals visit their clinics and at adoption locations (by animal shelters, animal welfare organizations, rescue organizations, and pet stores). The potential benefits of containing cats to an owner's property would need to be highlighted (such as the protection of cats from injury and the protection of native wildlife) as well as the different containment options available and advice on enrichment [e.g., (29)]. Owners' attitudes regarding their cat's “need” to roam would also need to be addressed. However, it is important to be aware that constraining the natural behaviors of cats, such as confining them indoors, have possible welfare implications due to boredom and inactivity. Therefore, suitable education on the needs of and appropriate enrichment, space, and housing requirements for contained cats is vital to allow them to express normal behaviors (24, 29, 81).

Changing the way cats are managed in New Zealand could also reduce the predation of native fauna species. Controlling cat roaming is not a popular idea in New Zealand, with only 5% of owned cats being housed indoors (3); however, the containment of other pets (e.g., dogs) is common practice and widely accepted, suggesting that an attitude change toward closer management of owned cats is possible. Predation and risk behaviors occurred both on and away from owners' properties in the current study (with the exception of venturing onto the road). Therefore, it is important to note that containing cats to their owner's properties will reduce, but not eliminate, their participation in these behaviors.

## Conclusion

This study is the first reporting on observations of predation and risk behaviors of owned cats in New Zealand using animal-borne cameras. Predatory behaviors were commonly displayed by the cats although no mammalian, amphibian, or avian species were preyed upon. Most of the identified prey species were invertebrates. Risk behaviors were commonly observed and included cats venturing onto the road; ingestion of plant material and water from potentially contaminated sources; altercations with other cats; and climbing on the edges of house, shed, or garage roofs, and into a storm water drain. Given the high frequency of risk behaviors in free-roaming owned cats, it is suggested that cat owners should be educated about strategies to minimize risk to their cats such as safely containing their cats or monitoring their time spent outdoors.

## Ethics Statement

This study was carried out in accordance with the recommendations of University of Auckland. This protocol was approved by the University of Auckland AEC (Auckland, New Zealand; Reference 001595).

## Author Contributions

SB, AD, GA, and JW oversaw the design and implementation of the study. SB performed the data collection. MG, SB, NG, and GA analyzed the data. SB, SZ, and MG wrote the paper. SB, SZ, MG, JW, AD, NG, and GA reviewed the manuscript.

### Conflict of Interest Statement

NG is employed by Wildland Consultants Ltd, a privately-owned ecological consultancy. JW is currently an employee of the New Zealand Companion Animal Council which is axillary to the New Zealand Companion Animal Trust. JW was not employed by the New Zealand Companion Animal Council when this research was conceived, funded, and conducted, and the funding body had no role in any aspects of the study design, data collection, analysis, interpretation of the data, the writing of the manuscript, or in the decision to publish the results. AD is the current Chair of the New Zealand Companion Animal Trust. AD was not in this role or employed by the New Zealand Companion Animal Council when this research was conceived, funded, and conducted, and the funding body had no role in any aspects of the study design, data collection, analysis, interpretation of the data, the writing of the manuscript, or in the decision to publish the results. The remaining authors declare that the research was conducted in the absence of any commercial or financial relationships that could be construed as a potential conflict of interest.

## Footnotes

^1^This information will be presented in another publication.

## Abbreviations

1080: Sodium fluoroacetate
D1: Day one
D2: Day two
D3: Day three
DOC: Department of Conservation
GPS: Global Positioning System
NZCAC: New Zealand Companion Animal Council
SH: Successful hunter.
